# Multiparity affects conduction properties of pelvic floor nerves in rabbits

**DOI:** 10.1002/brb3.1105

**Published:** 2018-09-21

**Authors:** Francisco Castelán, Kenia López‐García, Suelem Moreno‐Pérez, René Zempoalteca, Dora L. Corona‐Quintanilla, Mario I. Romero‐Ortega, Ismael Jiménez‐Estrada, Margarita Martínez‐Gómez

**Affiliations:** ^1^ Departamento de Biología Celular y Fisiología, Unidad Foránea Tlaxcala, Instituto de Investigaciones Biomédicas Universidad Nacional Autónoma de México Tlaxcala México; ^2^ Centro Tlaxcala de Biología de la Conducta Universidad Autónoma de Tlaxcala Tlaxcala México; ^3^ Maestría en Ciencias Biológicas Universidad Autónoma de Tlaxcala Tlaxcala México; ^4^ Departament of Bioengineering University of Texas at Dallas Dallas Texas; ^5^ Departamento de Fisiología, Biofísica y Neurociencias Centro de Investigación y de Estudios Avanzados del Instituto Politécnico Nacional Ciudad de México México

**Keywords:** bulbospongiosus muscle, micturition, myelin, pubococcygeus muscle, reproduction, urinary incontinence

## Abstract

**Introduction:**

Women often develop pelvic floor dysfunction due to damage to the pelvic musculature during childbirth; however, the effect on pelvic floor nerves function is less understood. This study used adult rabbits to evaluate the electrophysiological and histological characteristics of the bulbospongiosus (Bsn) and pubococcygeus nerves (Pcn) in multiparity.

**Methods:**

Compound nerve action potentials (CNAP) were compared between age‐matched nulliparous and multiparous animals and associated to the histological characteristics of myelinated axons from the Bsn and Pcn nerves. The *extensor digitorum longus* nerve (EDLn) was used as negative control. Data were analyzed with unpaired two‐tailed Student's *t* test or Mann–Whitney U test to determine significant differences between groups.

**Results:**

The onset and peak latencies, duration, and conduction velocity of the motor fibers in these pelvic nerves were not significantly different between nulliparous and multiparous animals. However, the peak‐to‐peak amplitude and area of the CNAP in both Bsn and Pcn were reduced in multiparous rabbits. Histology showed a higher percentage of axons with myelin disorganization caused by multiparity in these pelvic nerves. Together, the data indicate a reduction in the number of functional pelvic axons due to multiparity. As expected, no effect of parity was observed in the EDLn controls.

**Conclusions:**

Present findings demonstrated that multiparity affects myelination and consequently conduction properties in the small pelvic floor nerves.

## 1 INTRODUCTION

Pelvic floor muscles play a critical role in reproductive and excretory processes. Women are more prone than men to the onset of pelvic floor dysfunctions as the pelvic floor is more drastically impacted by pregnancy. Childbirth is a well‐recognized risk factor in developing pelvic organ prolapses and stress urinary incontinence (SUI). In both cases, there is compelling evidence indicating that the pelvic floor muscles are injured during pregnancy and parturition. Indeed, the weakening of pelvic floor muscles is considered the leading cause of SUI in women and might be related to impaired function of their motor innervation as the pudendal and perineal compound muscle action potential is reportedly affected (Olsen, Ross, & Stansfield, 2003). Furthermore, pudendal neuropathy has been associated with pelvic floor disorders including SUI in parous women (Sangwan et al., 1996; Snooks, Barnes, & Swash, 1984; Snooks, Swash, & Mathers, 1990), and a link between SUI and nerve injuries affecting the urethral viscerosomatic reflex has been reported (Aguiar et al., 2013). Consequently, a number of therapies to manage SUI, ranging from Kegel exercises to electrical stimulation of sacral and pudendal nerves, have been proposed as potential therapies to improve the function of pelvic floor musculature and its innervation (Elser, 2012).

Several studies involving primarily female rats have provided some understanding of the effects of injury in pelvic floor nerves and their contribution to SUI (Damaser, Broxton‐King, & Ferguson, 2003; Sajadi, Gill, & Damaser, 2010; Song et al., 2015). However, most of these studies have focused on the pudendal nerve, which branches distally into motor nerves, modulating thus more than one pelvic floor muscle including the urethral rhabdosphincter (Damaser et al., 2003; Pacheco, Martínez‐Gómez, & Whipple, 1989; Pastelín, Juárez, & Damaser, 2012; Song et al., 2015). Compared to rats, domestic rabbits have larger pelvic floor muscles, including perineal ones, which facilitates the electrophysiological evaluation of their innervations (Corona‐Quintanilla, Castelán, & Fajardo, 2009; Martínez‐Gómez et al., 2011; Martínez‐Gómez, Lucio, & Carro, 1997). In female rabbits, we have previously shown that the bulbospongiosus (Bsm, perineal) and pubococcygeus (Pcm, pelvic) muscles have antagonistic roles during micturition, as the Bsm contracts reflexively during the voiding while the Pcm is activated during the storage phase of micturition (Corona‐Quintanilla et al., 2009). These muscles are innervated by the bulbospongiosus (Bsn) and pubococcygeus nerves (Pcn) and are relatively smaller compared to the pudendal nerve (Cruz et al., 2017).

In female rabbits, the Bsn originates at the ischiorectal fossa, from the second branch of the pudendal nerve, and extends caudomedially toward the pubic bone, while the Pcn originates from the sacral S3 and S4 branches (Cruz et al., 2017). The anatomical location of Bsn and Pcn makes them susceptible to injuries due to pregnancy and childbirth, as indicated by the reportedly altered viscerosomatic reflexes, muscle injury, and impaired muscle contractility in multiparous rabbits (López‐García et al., 2016; López‐Juárez et al., 2018; Martínez‐Gómez et al., 2011). These alterations could be directly or indirectly related to deficiencies in the functional and anatomical properties of the pelvic and perineal innervation. This has been supported by a report that multiparity affects ganglia of the pelvic plexus located in the vicinity of Bsn and Pcn in rabbits (Castelán et al., 2013). Based on this information, we hypothesized that multiparity directly damages the pelvic floor nerves, and evaluated the electrophysiological and histological characteristics of Bsn and Pcn to determine the effect of multiparity on the compound nerve action potential (CNAP) parameters, and axon myelination in nulliparous and multiparous rabbits. The study confirmed the damage of the pelvic nerves due to multiparity.

## 2 MATERIAL AND METHODS

### 2.1 Animals

Age‐matched nulliparous (10.1 ± 0.4 months; N; *n* = 12) and multiparous (12.4 ± 0.6 months; M; *n* = 12) chinchilla‐breed female rabbits (*Oryctolagus cuniculus*) were housed in individual stainless‐steel cages (50 × 60 × 40 cm) at 24 ± 2ºC under artificial lighting (L: D 16:8, starting at 06:00 hr) in the vivarium of the Centro Tlaxcala de Biología de la Conducta, Universidad Autónoma de Tlaxcala. The animals were provided with pellet food (Purina, México) and water ad libitum. The Ethics Committee from the Instituto de Investigaciones Biomédicas, Universidad Nacional Autónoma de México, approved the experimental procedures.

Six‐month‐old nulliparous female rabbits mated with sexually experienced males and copulated 24 hr after the first three deliveries (Martínez‐Gómez et al., 2011). Thus, rabbits were pregnant and lactating for 20 days when pups were weaned. On the day of the fourth delivery, neonate pups were sacrificed to avoid lactation and to allow multiparas reaching a hormonal status quite similar to nulliparas as determined by the estradiol levels in serum (López‐García et al., 2013). Nulliparous animals were sacrificed when reached the age above mentioned and multiparous at postpartum day 20, with an urethane overdose (i.p.).

### 2.2 CNAP recordings

**Figure 1:**
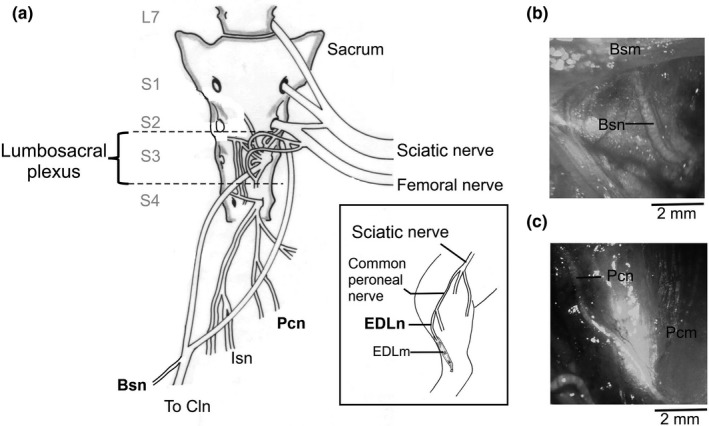
(a) Schematic view showing the anatomical location of bulbospongiosus (Bsn) and pubococcygeus (Pcn) nerves in the female rabbit; *dotted lines* delimit the lumbosacral plexus; the extensor digitorum longus nerve (EDLn) location is showed in the *Inset*. (b and c) representative photographs of dissected Bsn and Pcn

The Bsn and Pcn on the right side were dissected in six animals per group as recently described (Cruz et al., 2017). The animals were anesthetized with urethane (Sigma Chemical, USA; 0.9 g/kg; 20% in distiller water; i.p.) and fixed in a dorsal supine position, and the pelvic and perineal muscles were exposed. The Bsn and Pcn segments were dissected proximal to their muscle insertion (Figure 1a‐c). As control, a segment of the right *extensor digitorum longus* nerve (EDLn), a motor nerve located in the popliteal region of the leg, was exposed lateral to gastrocnemius muscle and dissected (Figure 1a, inset). The excised, Pcn, Bsn, and EDLn of each animal were transferred to a chamber filled with Krebs buffer solution (NaCl, 128 mM; KCI, 3 mM; CaCl_2_, 2 mM; MgSO_4_, 1 mM; NaHCO_3_, 21 mM; NaH_2_PO_4_, 0.5 mM; D‐glucosa, 30 mM, 95% O_2_, 5% CO_2_, pH 7) at 37°C.

The nerves were stimulated by wrapping silver wire electrodes connected to a constant current stimulator (Digitimer DS3) at one end and recording from suction electrodes made with glass capillaries filled with the Krebs saline solution and polished tips. The CNAP were evoked by the application of square current pulses at variable intensities trigger by a Grass stimulator (S48). The evoked responses were recorded by drawing two nerve segments into the suction electrodes connected to a grass amplifier (Grass 7P511) and to an oscilloscope (Tektronix TDS2024C) to visualize and record data. The stimulation train was ascendant with current graduate pulses (µA), 0.05 ms pulse duration at 1 Hz frequency. The activation threshold (1 × T) was defined as the minimum current that evoked a visible CNAP. Afterward, the current was gradually increased until reach the maximal CNAP responses (approximately 6 × T). It is important to note that the inversion of the polarity did not change any CNAP of the nerves.

The recorded CNAP were used to measure the maximal amplitude (A), considered as the voltage (mV) at the peak value of the CNAP; the peak latency (pl), calculated time elapsed from the stimulus artifact to the CNAP peak and the onset latency (ol), the time elapsed from the stimulus to the beginning of the CNAP multiplied by A and by 2; the CNAP area, calculated from [(pl‐ol)*A]*2; the CNAP duration, calculated from (pl‐ol)*2; and the peak conduction velocity, which was calculated dividing the length of the nerve section by the elapsed time from the stimulus to the CNAP‐component peak.

### 2.3 Nerve histology

An additional cohort of 6 animals per group was used to evaluate the histology of the Bsn, Pcn, and EDLn. The nerves were dissected and immediately fixed in Karnovsky buffer (200 mM sodium cacodylate, 25% glutaraldehyde, 1% paraformaldehyde, pH 7.3) during 24 hr at 4°C. The tissue was then immersed into 100 mM sodium cacodylate, pH 7.4, until embedded in epon resin. The tissue was incubated in 1% sodium tetroxide diluted in Zetterquist´s buffer (5% veronal acetate, pH 7.3–7.5) for 1 hr, washed three times with distilled water, and dehydrated with ascending concentrations of ethanol (70%, 80%, 90%, and 100%) 10 min each, at room temperature. The tissue was then washed two times for 15 min in a propylene oxide solution. Finally, nerve segments were immersed in epon‐acetonitrile mix solution 1:1 (v/v) during 1 hr, rinsed in epon‐acetonitrile mix solution 2:1 (v/v) for 1 hr, sank in pure epon for 16 hr in constant rotation, and transferred to inclusion templates at 60°C for 24 hr for resin polymerization.

The tissue was cut in 1 μm semithin transverse sections in an ultramicrotome (Leica Ultracut UCT) and stained with toluidine blue. Slides were analyzed using a light microscopy (Olympus CX31), and photomicrographs taken with an attached digital camera (Nikon DS‐Ri1). Axons that did not show a well‐defined circular form of the myelin sheet, that had myelin invaginations, myelin interruption, and/or apparent delamination of the myelin sheaths, were identified as “*axons with myelin disorganization*” and counted. The digital images of the nerves were divided into quadrants with a grid placed at the center of each fascicle. In the right superior quadrant, the total number of myelinated axons either normal or with myelin disorganization were counted and reported as percentage of the total number of axons in that quadrant.

### 2.4 Statistical analyses

Data are presented as the mean ± standard error of the mean (*SEM*), unless otherwise stated. The normality of data was assessed using a Kolmogorov–Smirnov test, and the means for each group analyzed with an unpaired two‐tailed Student's *t* test or Mann–Whitney *U* test. *p* < 0.05 values were considered statistical significant. Statistical analysis was performed using the GraphPad Prism 6.0e software (GraphPad).

## 3 RESULTS

Nulliparous and multiparous rabbits had similar body weights, 3.78 ± 0.18 and 3.46 ± 0.14 kg, respectively (*t* = 1.346, *p* = 0.208). The length of nerve segments used to record the CNAP responses were comparable in both groups (Bsn: 6.51 ± 0.21 vs. 6.76 ± 0.20 mm, U = 11, *p* = 0.27; Pcn: 6.58 ± 0.32 vs. 6.66 ± 0.16 mm, U = 16.5, *p* = 0.86; EDLn: 7.08 ± 0.08 vs. 7.02 ± 0.05 mm, U = 17.5, *p* = 1).

### 3.1 Evoked CNAP area and amplitude were reduced in the pelvic floor nerves of multiparous animals

**Figure 2:**
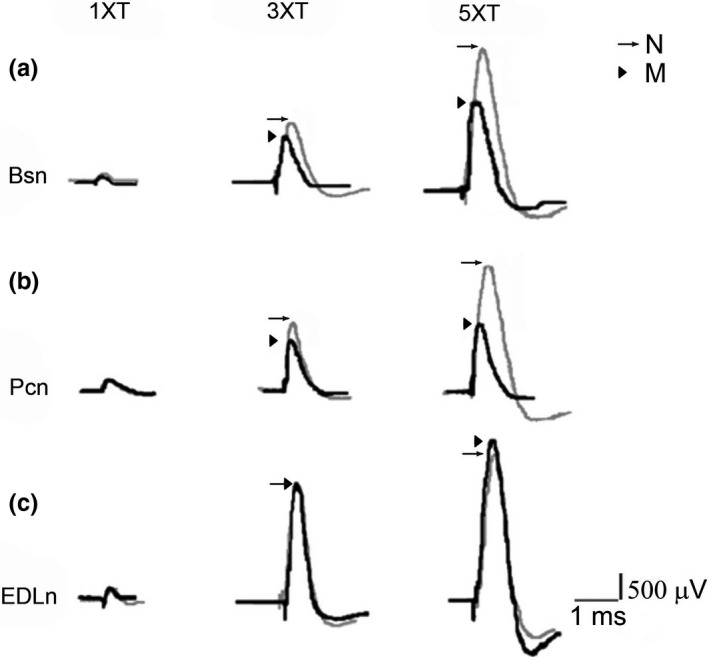
Multiparity affects specifically the CNAP evoked in Bsn and Pcn but not in the EDLn of rabbits. Representative CNAP traces induced by single current pulses applied with different strengths (1, 3 and 5 times threshold, ×T) to Bsn (a), Pcn (b), and EDLn (c) in nulliparous (N; *gray lines and arrows*) and multiparous rabbits (M, *black lines and arrowheads*). Traces represent the mean of 16 traces per nerve

The evoked CNAP in the Bsn and Pcn (Figure 2a,b), but not of the EDLn of multiparous animals was reduced compared to nulliparous rabbits (Figure 2c). Values for the onset and peak latencies, duration, and peak conduction velocity for Bsn, Pcn, and EDLn were not significantly different in nulliparous and multiparous rabbits (Tables 1, 2, 3).

**Table 1:** Latencies to the onset and peak of CNAP (ms) in bulbospongiosus, pubococcygeus, and EDL nerves

*Nerve*	Threshold (XT)	Onset latency (ms)				Peak latency (ms)			
		Nulliparous	Multiparous	*t* or *U* value	*p*	Nulliparous	Multiparous	*t* or *U* value	*p*
Bulbospongiosus	1	0.18 ± 0.02	0.17 ± 0.04	*t = *0.13	0.88	0.62 ± 0.13	0.50 ± 0.11	*t = *0.66	0.52
	2	0.18 ± 0.02	0.17 ± 0.04	*t = *0.13	0.89	0.62 ± 0.09	0.54 ± 0.08	*t = *0.61	0.55
	3	0.19 ± 0.03	0.17 ± 0.04	*t = *0.34	0.73	0.62 ± 0.09	0.54 ± 0.08	*t = *0.85	0.41
	4	0.20 ± 0.03	0.17 ± 0.04	*t = *0.47	0.64	0.70 ± 0.12	0.56 ± 0.07	*t = *0.99	0.34
	5	0.21 ± 0.04	0.17 ± 0.04	*t = *0.59	0.56	0.70 ± 0.11	0.57 ± 0.06	*t = *0.98	0.34
	6	0.22 ± 0.05	0.17 ± 0.04	*t = *0.71	0.49	0.68 ± 0.09	0.57 ± 0.06	*t = *1.01	0.33
Pubococcygeus	1	0.15 ± 0.02	0.14 ± 0.03	*t = *0.16	0.87	0.55 ± 0.06	0.45 ± 0.06	*t = *1.03	0.32
	2	0.13 ± 0.02	0.15 ± 0.03	*t = *0.40	0.69	0.50 ± 0.07	0.46 ± 0.06	*t = *0.37	0.71
	3	0.13 ± 0.02	0.14 ± 0.03	*U = *17.0	0.87	0.49 ± 0.07	0.47 ± 0.07	*t = *0.21	0.83
	4	0.13 ± 0.02	0.14 ± 0.03	*U = *17.5	>0.99	0.54 ± 0.07	0.47 ± 0.07	*t = *0.65	0.52
	5	0.11 ± 0.01	0.11 ± 0.01	*U = *17.5	0.99	0.55 ± 0.07	0.45 ± 0.06	*t = *1.06	0.31
	6	0.11 ± 0.01	0.13 ± 0.03	*U = *16.5	0.08	0.56 ± 0.06	0.44 ± 0.06	*t = *1.29	0.22
EDL	1	0.20 ± 0.05	0.18 ± 0.03	*t = *0.38	0.70	0.68 ± 0.09	1.00 ± 0.12	*t = *0.02	0.97
	2	0.19 ± 0.03	0.16 ± 0.01	*t = *0.63	0.53	0.62 ± 0.06	0.96 ± 0.13	*U = *18.0	0.09
	3	0.20 ± 0.03	0.15 ± 0.01	*t = *1.35	0.20	0.63 ± 0.09	0.87 ± 0.03	*t = *0.41	0.68
	4	0.20 ± 0.03	0.15 ± 0.01	*t = *1.35	0.20	0.65 ± 0.10	0.87 ± 0.03	*t = *0.53	0.60
	5	0.20 ± 0.03	0.19 ± 0.02	*t = *0.40	0.69	0.64 ± 0.10	0.84 ± 0.02	*U = *18.0	0.69
	6	0.20 ± 0.03	0.19 ± 0.02	*t = *0.40	0.69	0.66 ± 0.11	0.87 ± 0.03	*U = *18.0	>0.99

Notes

CNAP, Compound nerve action potential; EDL, Extensor digitorum longus; XT, *x* times Threshold.

Data are means ± *SEM* from 16 measurements per rabbit per group (*n* = 6 animals per group). Either Student´s *t* test or Mann–Whitney *U* test was done for comparisons between nulliparous and multiparous rabbits.

**Table 2:** CNAP duration (ms) in bulbospongiosus, pubococcygeus, and EDL nerves

Nerve	Threshold (XT)	Nulliparous	Multiparous	*t* or *U* value	*p*
Bulbospongiosus	1	0.88 ± 0.22	0.65 ± 0.15	*t = *0.84	0.41
	2	0.88 ± 0.14	0.73 ± 0.11	*t = *0.78	0.44
	3	0.90 ± 0.33	0.73 ± 0.11	*t = *0.96	0.35
	4	1.00 ± 0.19	0.77 ± 0.09	*U = *16.0	0.80
	5	0.99 ± 0.16	0.80 ± 0.79	*U = *15.5	0.74
	6	0.93 ± 0.08	0.80 ± 0.07	*t = *1.13	0.28
Pubococcygeus	1	0.80 ± 0.14	0.62 ± 0.10	*t = *0.99	0.34
	2	0.72 ± 0.15	0.62 ± 0.10	*t = *0.56	0.58
	3	0.72 ± 0.15	0.82 ± 0.14	*t = *0.35	0.72
	4	0.82 ± 0.14	0.64 ± 0.13	*t = *0.88	0.39
	5	0.88 ± 0.12	0.68 ± 0.09	*t = *0.24	1.24
	6	0.9 ± 0.12	0.63 ± 0.08	*t = *1.29	0.10
EDL	1	0.96 ± 0.10	1.00 ± 0.12	*t = *0.24	0.80
	2	0.86 ± 0.07	0.96 ± 0.13	*U = *17.0	0.93
	3	0.85 ± 0.11	0.87 ± 0.03	*t = *0.15	0.87
	4	0.88 ± 0.14	0.87 ± 0.03	*t = *0.09	0.92
	5	0.87 ± 0.14	0.84 ± 0.02	*U = *16.0	0.79
	6	0.90 ± 0.16	0.87 ± 0.03	*t = *0.19	0.84

CNAP, Compound nerve action potential; EDL, Extensor digitorum longus; XT, *x* times Threshold.

Data are means ± *SEM* from 16 measurements per rabbit per group (*n* = 6 animals per group). Either Student´s *t* test or Mann–Whitney *U* test was done for comparisons between nulliparous and multiparous rabbits.

**Table 3:** Peak conduction velocity of CNAP (m/s) in bulbospongiosus, pubococcygeus, and EDL nerves

Nerve	Threshold (XT)	Nulliparous	Multiparous	*t* or *U* value	*p*
Bulbospongiosus	1	12.89 ± 2.29	17.52 ± 3.62	*t = *0.88	0.39
	2	11.75 ± 1.68	14.49 ± 2.59	*t = *1.36	0.20
	3	10.84 ± 1.03	14.21 ± 2.24	*t = *1.29	0.22
	4	10.38 ± 1.24	13.12 ± 1.70	*t = *1.29	0.22
	5	10.19 ± 1.15	12.56 ± 1.42	*t = *1.29	0.22
	6	10.12 ± 0.95	12.56 ± 1.42	*t = *1.42	0.18
Pubococcygeus	1	12.94 ± 2.01	16.46 ± 2.81	*t = *1.01	0.33
	2	14.46 ± 1.90	16.23 ± 2.85	*t = *0.51	0.61
	3	14.98 ± 2.33	10.25 ± 2.90	*t = *0.34	0.74
	4	13.42 ± 1.78	16.25 ± 2.90	*t = *0.82	0.42
	5	12.83 ± 1.45	15.94 ± 1.98	*t = *1.26	0.23
	6	12.43 ± 1.33	16.22 ± 1.97	*t = *1.59	0.14
EDL	1	11.53 ± 1.78	11.16 ± 1.34	*t = *0.16	0.87
	2	12.18 ± 1.51	11.46 ± 0.96	*t = *0.40	0.69
	3	12.61 ± 2.05	11.96 ± 0.55	*t = *0.30	0.76
	4	12.48 ± 2.12	11.45 ± 0.52	*t = *0.24	0.81
	5	12.91 ± 2.47	11.56 ± 0.32	*t = *0.53	0.60
	6	12.78 ± 2.53	11.35 ± 0.50	*U = *17.0	0.93

CNAP, Compound nerve action potential; EDL, Extensor digitorum longus; XT, *x* times Threshold.

Data are means ± SEM from 16 measurements per rabbit per group (*n* = 6 animals per group). Either Student´s *t* test or Mann–Whitney *U* test was done for comparisons between nulliparous and multiparous rabbits.

**Figure 3:**
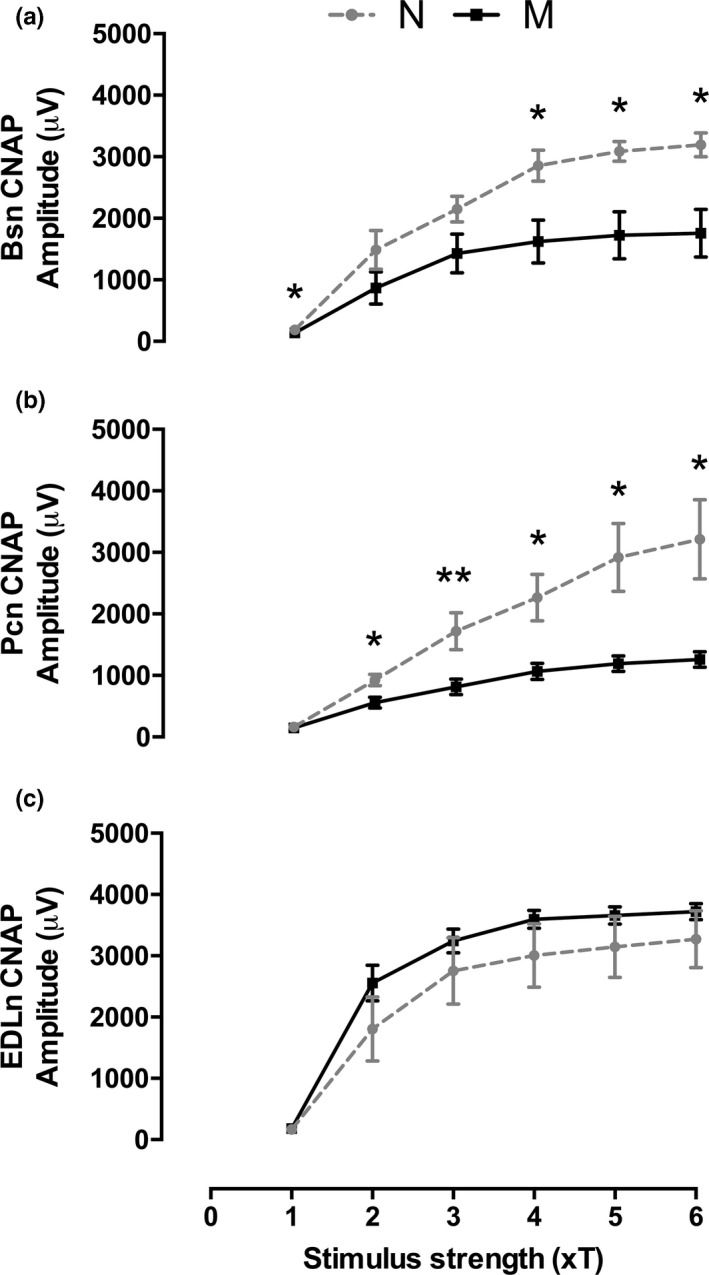
Multiparity decreases the CNAP amplitude of perineal and pelvic nerves in rabbits. Data are means ± *SEM* (*n* = 6 per group) of the CNAP amplitude provoked by electrical current pulses of gradually increased strength (from 1 to 6 times threshold, ×T) applied to the Bsn (a), Pcn (b), and EDLn (c) from nulliparous (N) and multiparous (M) rabbits. *p < 0.05; **p < 0.01

The amplitude of the evoked CNAP in the Bsn in multiparous was reduced compared to that of nulliparous rabbits: 1 × *T*:* t* = 2.489, *p* = 0.032; 4 × *T*:* U* = 3, *p* = 0.019; 5 × *T*:* t* = 3.29, *p* = 0.008; 6 × *T*:* t* = 3.288, *p* = 0.008 (Figure 3a). Similar reductions in CNAP amplitude were observed in the Pcn: 2 × *T*:* t* = 2.82, *p* = 0.018; 3 × *T*:* U* = 1, *p* = 0.004; 4 × *T*:* t* = 2.99, *p* = 0.014; 5 × *T*:* t* = 3.03, *p* = 0.015; 6 × *T*:* t* = 2.99, *p* = 0.014 (Figure 3b). These changes were specific for the pelvic floor nerves as the amplitude on CNAP evoked in the EDLn was comparable in the two groups of animals (*p* > 0.05; Figure 3c).

**Figure 4:**
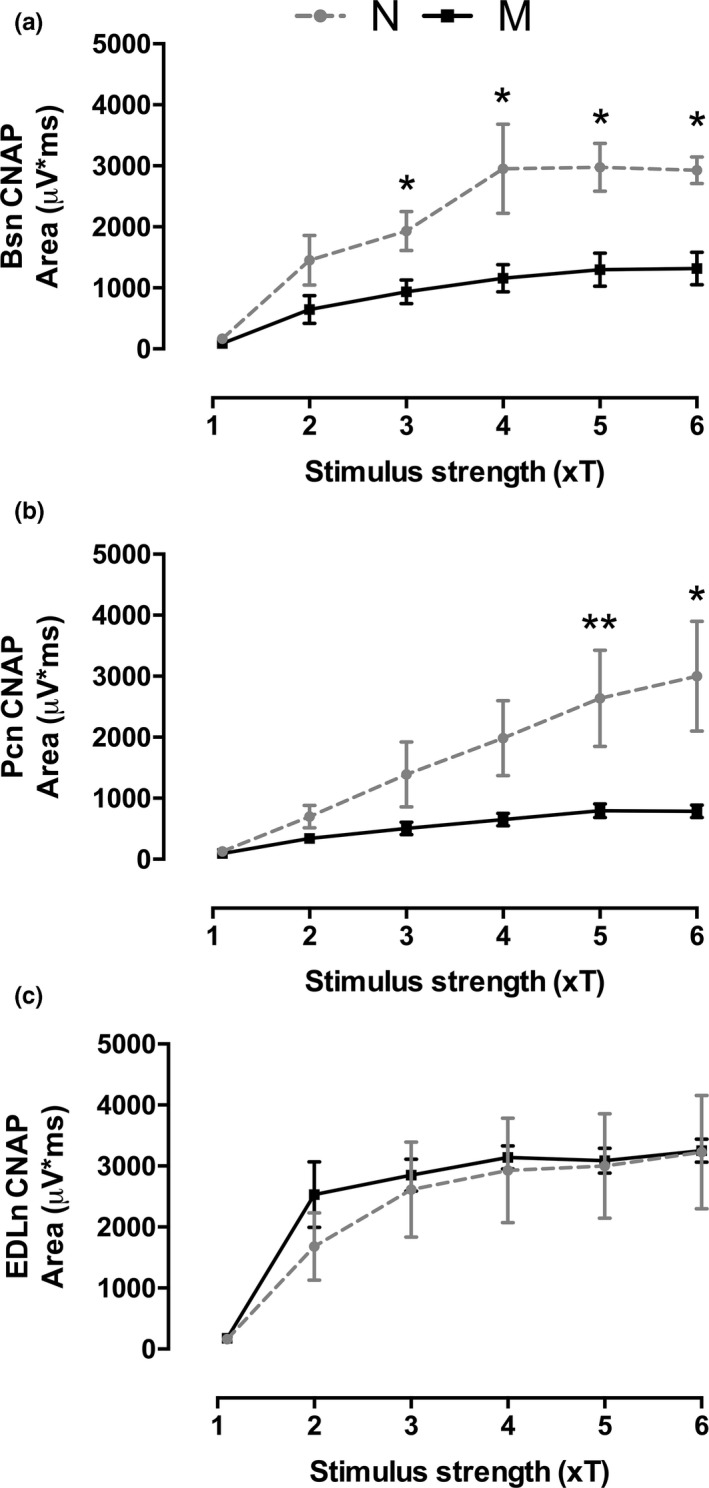
Multiparity decreases the CNAP area of perineal and pelvic nerves in rabbits. Data are means ± *SEM* (*n* = 6 per group) of the CNAP area evoked in Bsn (a), Pcn (b), and EDLn (c) from nulliparous (N) and multiparous (M) rabbits. *p < 0.05; **p < 0.01

In multiparous animals, the CNAP area of the Bsn was significantly reduced at 3 × *T*: 2.66, *p* = 0.024; 4 × *T*:* t* = 2.35, *p* = 0.041; 5 × *T*:* t* = 3.51, *p* = 0.006 and; 6 × *T*:* t* = 4.67. *p* = 0.001 compared to that in nulliparous rabbits (Figure 4a). The Pcn was also significantly reduced in these animals at 5 × *T*:* U* = 0, *p* = 0.002, and 6 × *T t* = 2.44, *p* = 0.035 (Figure 4b). Multiparity did not affect the CNAP area when measured in the EDLn (Figure 4c).

#### 3.1.1 Multiparity affected myelinated axons of the Bsn and Pcn but not of EDLn

**Figure 5:**
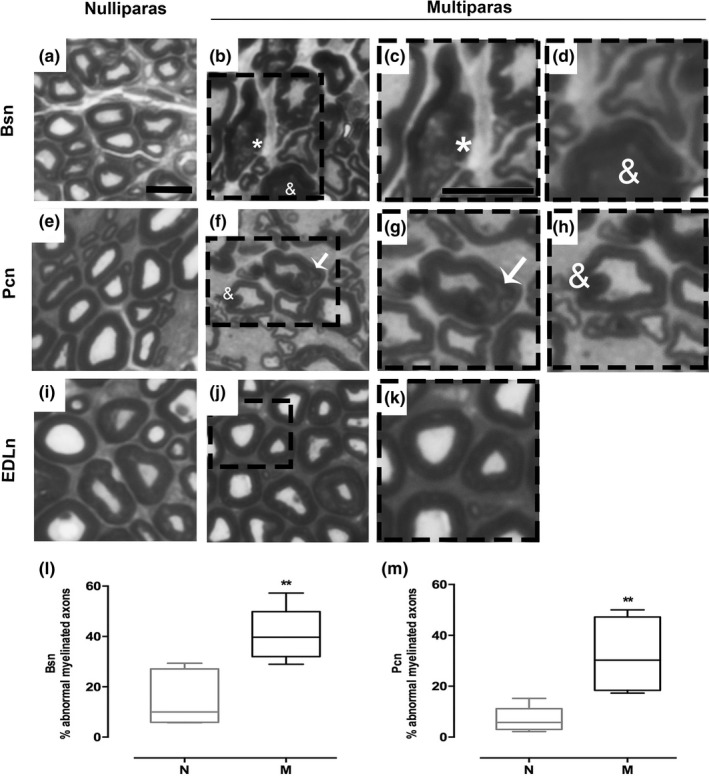
Multiparity increases the occurrence of axons with myelin disorganization in perineal and pelvic nerves of rabbits. Representative photomicrographs from Bsn (a–c), Pcn (d–f), and EDLn (g–i) transverse sections stained with Toluidine blue from nulliparas (N) and multiparas (M). Data are medians ±minimal to maximal values for Bsn (j) and Pcn (k). Symbols indicate abnormal myelinated fibers: myelin disruption (*asterisk*), invagination (*ampersand*), and apparent separation of the myelin sheaths (*arrow*). Scale bar, 10 μm

Evaluation of toluidine blue‐stained semithin sections showed remarkable differences in the appearance of myelinated axons between nulliparous and multiparous rabbits (Figure 5a‐k). Axons in the Bsn of nulliparas showed compact and well‐defined myelin sheets (Figure 5a). In contrast, several axons showed myelin disorganization including circumferential cleavage and infolded loops in multiparous animals (Figure 5b‐d). The Pcn was similarly affected by multiparity, as nulliparous animals had axons with regular, well‐defined shapes, and compact myelin (Figure 5e), contrasting with nerves in multiparous rabbits, that showed phagocytized myelin and abnormal myelination (Figure 5f‐h). In contrast, no axons with myelin disorganization were observed in the EDLn of both groups of animals (Figure 5i‐k). As percentage of normal axons, multiparous animals compared to nulliparours showed an increase in myelin disorganization in the Bsn (14.6 ± 4.3 vs. 41% ± 4.3%; *U* = 1, *p* = 0.0043; Figure 5k) and the Pcn (7 ± 2 vs. 31.9% ± 7.6%; *U* = 0, *p* = 0.0043; Figure 5l).

Importantly, the number of sampled myelinated axons in multiparous and nulliparous rabbits, Bsn (339  39 vs. 279  56; *t* = 0.873, *p* = 0.403) and Pcn (160  45 (*n* = 6) vs. 100  34; t = 0.965, *p* = 0.363 (*n* = 4)), was not significantly different.

## 4 DISCUSSION

Present findings demonstrate that multiparity significantly decreased the amplitude and area of the CNAP evoked in the pelvic Bsn and Pcn motor nerves. In contrast, the onset and peak latencies, duration, and conduction velocity of the motor fibers in these pelvic nerves were not significantly different between nulliparous and multiparous animals. None of variables measured from the CNAP evoked in the EDLn were affected by multiparity. This result was consistent with a higher percentage of axons with myelin disorganization in Bsn and Pcn from multiparous animals.

Twenty days after the fourth delivery, CNAP parameters as the latencies to the onset and to the peak evoked in Bsn and Pcn of nulliparas and multiparas did not differ. These results contrast with those from clinical studies reporting an increase in the pudendal nerve motor terminal latency (PNMTL) in patients with EAS and/or EUS dysfunction after parturition (Aguiar et al., 2013; Sangwan et al., 1996; Snooks et al., 1984, 1990 ; Tetzschner, Sørensen, & Lose, 1997). However, the different methodological approaches between ours and the last studies offer a possible explanation to this discrepancy. The present study used rabbit nerves ex vivo, and the clinical studies evaluated women with fecal or incontinence urinary of varied duration or parous women on 12 weeks postpartum. Furthermore, the nerves evaluated in both studies were different; the pudendal nerve was examined clinically, whereas the Bsn and Pcn were examined in multiparous rabbits 20 days after the fourth delivery. It is also possible that a more drastic impair of the EAS was involved in the PNMTL cases (Snooks et al., 1990).

In this study, we reported conduction velocities for both Bsn and Pcn higher than 10 m/sec, which is in agreement with values estimated for myelinated fibers of the pelvic nerve of rats (Nakayama, Noda, & Hotta, 1998). In the study conducted for Nakayama and colleagues, the shorter nerve segment used could have impede to discriminate the fast large myelinated fibers, which is supported herein from data gathered from EDLn (Li & Shi, 2007). We noted that multiparity does not affect the CNAP‐peak conduction velocities of Bsn and Pcn in rabbits, suggesting that multiparity does not significantly affect the conduction velocity of the majority of myelinated fibers (e.g., fiber diameter, internode distance, nodal area, etc. (Waxman, 1980)), as has been reported for myelinated fibers in the pelvic nerves of aging rats (Nakayama et al., 1998).

Multiparity decreases the amplitude and area of CNAP of the Bsn and Pcn, to 40% and 70% observed in nulliparous animals, respectively. Considering that this value represents the extent in which summed action potentials are propagated along the nerve; this finding suggests that partial damage occurred to some axons in these pelvic nerves due to multiparity. This notion is consistent with the observed myelin disorganization in the toluidine blue‐stained semithin sections of both nerves. Altogether, these data support the interpretation that the observed focal demyelination in these nerves caused by multiparity impairs the conduction of the depolarization signal in the damaged axons, resulting in functional gaps between nodes of Ranvier (Robinson, 2000). Certainly, the ultrastructural evaluation of the Bsn and Pcn using electron microscopy is warranted to further define the specific types of myelin damaged that occur as a result of parturition. It is known that pudendal nerve crush injury during vaginal distention at delivery results in abnormal nerve fascicles around the urethra, reducing the leak point pressure in female rats (Damaser et al., 2003). Overall, the impaired propagation of action potentials of the Bsn and Pcn could explain the desynchronized activity of Bsm and Pcm during micturition in multiparous rabbits (Martínez‐Gómez et al., 2011). This could be also influenced by the time course of muscle degeneration and the possible regeneration of Pcm and Bsm and changes in contractile properties of both muscles in multiparous rabbits (López‐García et al., 2016; López‐Juárez et al., 2018).

The observed alterations in area and amplitude of the CNAP and myelinated fibers in Bsn and Pcn caused by multiparity could be also linked to the anatomical position of these muscle in pelvic floor of female rabbits (Cruz et al., 2017). The successive passage of the fetus through the perineal vagina during each delivery likely compresses the Bsn against the sciatic arch (Cruz et al., 2017), which would be exacerbated by consecutive deliveries. The Pcn, instead, if formed by several rami originated from the sacral segments S3 and S4 and extends along other pelvic structures and viscera. During parturition, this nerve is likely to be stretched during the elevation of the tail to reduce the pelvic vagina pressure, facilitating the expulsion of pups (López‐Juárez et al., 2018; Martínez‐Gómez et al., 1997).

Clinical and basic science studies support that labor trauma induces damage to pelvic floor nerves, which leads to the onset of pelvic floor disorders including fecal and urinary incontinence (Aguiar et al., 2013; Snooks et al., 1984, 1990 ; Tetzschner et al., 1997). This is commonly accepted as one of the frequent causes of EAS and EUS disabilities (Olsen et al., 2003). Functional and histological evidence showed in this report indicates that multiparity in rabbits partially impairs the function of Bsn and Pcn, persisting for 20 days after delivery. This damage seems correlated to the disorganized reflex myographic activity of the Bsm and Pcm that has been reported in animals with impaired urodynamic function (Martínez‐Gómez et al., 2011). To our knowledge, this is the first study reporting the direct nerve injury to small nerves of pelvic floor, generated as a consequence of multiparity. Further studies, however, are needed to address the possible extent and duration of recovery of these damaged myelinated axons in Bsn and Pcn.

An important limitation of this study is the lack of information on the possible effects of parturition on other axon types including medium and slow conducting B‐ and C‐fibers. The relatively low current used in this study were below the activation threshold for these fibers. In rabbits, the Bsm assists in voiding while the Pcm assist in storage of urine (Corona‐Quintanilla et al., 2009). Given the partially affected nature of Bsn and Pcn concurrent with the disorganized activation of Bsm and Pcm, and urodynamic alterations observed in multiparous rabbits (Corona‐Quintanilla et al., 2009; Martínez‐Gómez et al., 2011), further basic science studies should analyze different strategies aimed to recovery the function of the pelvic floor muscles. Such studies would be valuable in determining whether the recovery or activation of the damaged pelvic nerves may be a viable therapeutic target for the treatment of SUI.

In summary, our present findings indicate that multiparity reduces the CNAP amplitude and area of the Bsn and Pcn in rabbits. These changes were associated with a high percentage of abnormal myelinated axons, and underlie the importance of the pelvic floor neuromuscular damage, as a clinical target for the treatment of the dysfunctional pelvic floor, including SUI.

## CONFLICT OF INTEREST

The authors declare no conflict of interests.
